# Effectiveness of a psychoeducation group intervention conducted by primary healthcare nurses in patients with depression and physical comorbidity: study protocol for a randomized, controlled trial

**DOI:** 10.1186/s12913-019-4198-7

**Published:** 2019-06-26

**Authors:** Rocío Casañas, Jaume Martín Royo, Maria Isabel Fernandez-San-Martín, Antonia Raya Tena, Jacobo Mendioroz, Glòria Sauch Valmaña, Roser Masa-Font, Marc Casajuana-Closas, Eva María Fernandez Linares, Cèlia Cols-Sagarra, Susana Gonzalez Tejón, Quintí Foguet-Boreu, Luis Miguel Martín Lopez

**Affiliations:** 1 Research Department, Associació Centre Higiene Mental Les Corts, Barcelona, Spain; 2 Fundació Institut Universitari per a la recerca a l’Atenció Primària de Salut Jordi Gol i Gurina (IDIAPJGol), Barcelona, Spain; 3 Centre d’Urgències d’Atenció Primària (CUAP) Casernes, Institut Català de la Salut, Barcelona, Spain; 4 Unitat de Suport a la Recerca Barcelona Ciutat, Fundació Institut Universitari per a la recerca a l’Atenció Primària de Salut Jordi Gol i Gurina (IDIAPJGol), Barcelona, Spain; 5 Unitat Docent Multiprofesional Gerència Territorial Barcelona, Institut Català de la Salut, Barcelona, Spain; 6 Centre d’Atenció Primària Raval Nord, Institut Català de la Salut, Barcelona, Spain; 7 Unitat de Suport a la Recerca Catalunya Central, Fundació Institut Universitari per a la recerca a l’Atenció Primària de Salut Jordi Gol i Gurina (IDIAPJGol), Barcelona, Spain; 8 Centre d’Atenció Primària Besos, Institut Català de la Salut, Barcelona, Spain; 9 Centre d’Atenció Primària Raval Sud, Línea Pediàtrica, Institut Català de la Salut, Barcelona, Spain; 10 Centre d’Atenció Primària Martorell Rural, Institut Català de la Salut, Barcelona, Martorell Spain; 11 Centre d’Atenció Primària Raval Sud, Institut Català de la Salut, Barcelona, Spain; 12 Instituto de Neuropsiquiatria y Adicciones del Parc de Salut del Mar (INAD), Consorci Parc de Salut Mar, Barcelona, Spain; 13 Universitat Autónoma de Barcelona, Bellaterra, Cerdanyola del Valles Spain; 14 Health Promotion in Rural Areas Research Group (PRoSaARu), Gerència Territorial de la Catalunya Central, Catalan Health Institute, Sant Fruitós de Bages, Barcelona, Spain

**Keywords:** Depression, Primary healthcare, Chronic physical illness, Nurses, Psychoeducation

## Abstract

**Background:**

Depressive disorders are the third leading cause of consultation in primary care, mainly in patients with chronic physical illnesses. Studies have shown the effectiveness of group psychoeducation in reducing symptoms in depressive individuals. Our primary aim is to evaluate the effectiveness of an intervention based on a psychoeducational program, carried out by primary care nurses, to improve the remission/response rate of depression in patients with chronic physical illness. Secondarily, to assess the cost-effectiveness of the intervention, its impact on improving control of the physical pathology and quality of life, and intervention feasibility.

**Methods/design:**

A multicenter, randomized, clinical trial, with two groups and one-year follow-up evaluation. Economic evaluation study.

**Subjects:**

We will assess 504 patients (252 in each group) aged > 50 years assigned to 25 primary healthcare centers (PHC) from Catalonia (urban, semi-urban, and rural). Participants suffer from major depression (Beck depression inventory: BDI-II 13–28) and at least one of the following: type 2 diabetes mellitus, chronic obstructive pulmonary disease, asthma, and/or ischemic cardiopathy. Patients with moderate/severe suicide risk or severe mental disorders are excluded. Participants will be distributed randomly into the intervention group (IG) and control (CG).

**Intervention:**

The IG will participate in the psychoeducational intervention: 12 sessions of 90 min, once a week led by two Primary Care (PC) nurses. The sessions will consist of health education regarding chronic physical illness and depressive symptoms.

**Main measurements:**

Clinical remission of depression and/or response to intervention (BDI-II).

**Secondary measurements:**

Improvement in control of chronic diseases (blood test and physical parameters), drug compliance (Morinsky-Green test and number of containers returned), quality of life (EQ-5D), medical service utilization (appointments and hospital admissions due to complications), and feasibility of the intervention (satisfaction and compliance). Evaluations will be blinded, and conducted at baseline, post-intervention, and 12 months follow-up.

**Discussion:**

Results could be informative for efforts to prevent depression in patients with a chronic physical illness.

**Trial registration:**

NCT03243799 (registration date August 9, 2017)

## Background

Depression is considered a major public health issue in industrialized societies [1], and has been associated with greater morbidity and mortality, and increased healthcare utilization and costs [2–4]. In primary care, depression is one of the most frequent causes of consultation [5] and is particularly common in patients with physical illnesses. The prevalence of depression worldwide is 4.4%, in Spain it represents 5.2% [6] reaching up to 30% in populations with chronic physical conditions when taking into account symptoms and disorders [2]. For instance, in diabetic patients the risk of depression is twice that of the general population [2]. According to the World Health Organization (WHO) by 2030 it will be the principal cause of disability in the world [7].

The concomitance of physical illness and depression not only decreases the patient’s response to anti-depressive treatment but also worsens control of their organic pathologies, results in unhealthy habits (diet and exercise), and leads to lower treatment compliance [8]. As a consequence, the economic burden of healthcare is greater in such individuals with depression than those without [7]. For instance, patients with depression and chronic obstructive pulmonary disease (COPD) can present exacerbations of common respiratory symptoms which require a greater number of visits to the emergency room and hospitalization [9]. It is thus becoming increasingly necessary to design strategies aimed at detecting and treating depression in patients with associated physical comorbidity in order to reduce depressive symptoms and disorders, and improve general health.

A number of studies has shown that preventive interventions, particularly stepped-care ones, can lead to a reduction of up to 25% in the rate of depression in diseases such as diabetes [10]. Most of the research, however, has been focused on reducing depressive symptoms and disorders, and there is less directed at obtaining health benefits regarding the parameters of the associated medical illness [11, 12]. According to the literature, there are greater benefits to be had from intervening rather than not [10,11,]. Nevertheless, there is some controversy over whether specific and specialized interventions are better than more general ones such as health education regarding the associated chronic pathologies [13, 14]. As an example, the DIAMOS study reported that behavioral techniques led to both a decrease in depressive symptoms and a reduction in blood glucose levels [15].

With regard to psychoeducation, it has been demonstrated that it is an effective therapy in the treatment of depression in adults [16, 17] as it decreases depressive symptoms and risk of relapse/recurrence, and improves treatment compliance [17–19]. Adherence to psychoeducation interventions is high, according to some authors, with a reported attendance of 73–87% in all the group sessions [18, 20]. Moreover, such therapy could be carried out in primary care by community nurses with previous training [18, 20].

We present a study protocol of a randomized controlled trial aimed at evaluating the effectiveness of a psychoeducation group intervention carried out by primary care nurses in patients with depression and chronic physical illness (diabetes, COPD, asthma and/or ischemic cardiopathy). The main objective is to evaluate whether such an intervention improves the rate of remission and response of depression in these patients. Secondary ones include assessing the cost-effectiveness and feasibility of the intervention, its efficacy in improving control of the physical pathology, and its impact on health-related quality of life (HRQoL).

The primary hypothesis is that in patients with chronic physical disease pyschoeducation group therapy provided by primary care nurses can achieve a greater rate of remission and response of depression than habitual clinical practice.

## Methods/design

### Study design

A randomized, multi-center, clinical trial composed of two groups with blinded response variables and a one-year follow-up. A cost-effectiveness study will also be performed. When each primary healthcare center (PHC) has a sample of 24 patients these are randomly allocated to either an intervention group (IG) or a control (CG). Data will be collected at baseline, and at 3 and 12 months post-intervention.

### Sample

The participants are patients aged > 50 years assigned to PHC from different locations: Barcelona city (1,200,066), the central area of Catalonia (192,000), and the southern area (132,000). Such a territorial representation provides patients from urban, semi-urban, and rural areas.

Inclusion criteria are: 1) the presence of at least one of the following physical diseases: diabetes mellitus type 2, COPD, asthma, and ischemic cardiopathy; 2) a score > 12 on the Beck Depression Inventory score (BDI-II) [21, 22] Spanish version adapted by Sanz et al. [23–25], and confirmation of major depression according to DSM-IV criteria with the Mini International Neuropsychiatric Interview (MINI); 3) adherence to a one-year follow-up with the same PHC team; 4) signed informed consent.

Exclusion criteria are: 1) diagnosis of dementia or moderate/elevated cognitive decline (5 or more errors on the Pfeiffer scale); 2) major depressive disorder with psychotic symptoms or other serious psychiatric comorbidities; 3) elevated/moderate risk of suicide (a score of ≥6 on the MINI scale); 4) drug of abuse dependence disorders (including alcohol); 5) physical illness at an advanced stage; 6) inability to attend the PHC; 7) under psychological therapy from the Community Mental Health Team; 8) inability to understand Spanish/Catalan.

Patients receiving anti-depressive/anxiolytic treatment are not excluded. Their data are collected and considered as covariables.

### Sample size

A power analysis will be conducted to determine the appropriate sample size. A previous study of psychoeducation programs [18] reported a remission rate at 9 months follow-up of 40% in the IG and 26% in the CG. Assuming a 5 and 20% alpha and beta risk, respectively, 189 patients are required for each group. Due to a number of reasons, an approximately 25% dropout rate is expected, consequently, 252 participants per group need to be recruited (total *n* = 504). In addition, 25 PHC are required each with a mean of 20 patients (IG *n* = 10, CG *n* = 10).

### Intervention

#### Description of the psychoeducation group intervention

In order to homogenize the interventions, the research group has developed a protocol of 12 weekly sessions lasting 90 min led by two primary care nurses. Each group is made up of 8–12 participants [26]. The sessions will be held on PHC premises which have the necessary space and equipment.

The objectives of the 12 sessions are depicted in Table 1. The program provides: 1) health education about chronic pathology and depressive symptoms; 2) information on the relationship between depressive symptoms and chronic pathology; 3) health education regarding: diet, physical exercise, sleep, pleasant activities, social skills, pharmacological treatment, and adherence to treatment; 4) breathing techniques; 5) problem solving, behavioral activation, and cognitive-behavioral perspective on depression; and 6) self-esteem and assertiveness [26].

**Table 1:** Content of the psychoeducational group program

lSessions	Objectives
1	• First contact with the group • Identification of depressive symptoms • Information on chronic pathology versus chronicity • Information on the relationship between depressive symptoms and chronic pathology • Breathing techniques
2	• Behavioural Activation I: Concern and problems.
3	• Behavioural Activation II: Concern and problems.
4	• How to take care to advance I ➢ Healthy diet ➢ Motivation for change (resources and difficulties)
5	• How to take care to advance II ➢ Physical exercise ➢ Pleasant activities, social skills
6	• How to take care to advance III ➢ Sleep ➢ Therapeutic compliance
7	• Problem solving I
8	• Problem solving II
9	• Self-esteem and assertiveness
10	• Cognitive-behavioural perspective I
11	• Cognitive-behavioural perspective II
12	• Group farewell • Final evaluation

To enhance the active role of the patient, each session is accompanied with homework. The participants are free to continue under pharmacological treatment.

#### Description of the control group

Members of the CG receive their usual treatment, that is to say, they can consult their family doctor/nurse as needed with no set pattern. The appointments last from 10 to 20 min and for both groups the healthcare professionals use their own criteria (usual clinical practice) regarding the chronic pathology and depressive symptoms.

The nurses receive prior training based on the intervention protocol [26] including the content of the 12 sessions. It consists of 20 h (10 theory and 10 practice) and is given by psychologists and nurses specialized in mental health.

### Recruitment process

The recruitment process for the trial began in September 2017. All eligible PHC located in Barcelona city, the central area of Catalonia (Bagés, Berguedá, and Solsonés) and the southern area (Baix Llobregat Nord) have been contacted and encouraged to participate in the study. The PHC who express interest receive a team presentation, explanatory dossiers, and informed consent papers. Those who wish to take part are required to consecutively recruit 16–24 patients who, after meeting inclusion criteria and signing the informed consent, will be randomized into two equally sized groups (IG and CG).

Different groups of healthcare workers will be involved in the field work:

- Recruiters: the patient’s assigned doctor and/or nurse.
- Nurses leading the psycho-educational groups, two per PHC.
- Evaluators blinded to the groups who carry out interviews at baseline, and at 3 months, and 12 months post-intervention.
- An external researcher in charge of patient randomization which is performed once all the necessary number of patients have signed the consent forms. The data gathering phases are explained in the work plan section.

All outcome variables will be assessed three times: prior to study commencement (baseline), after 3 months, and at 12 months after inclusion in the individual data collection sessions. Information about data collection and intervention programme timeline are detailed in the Table 2.

**Table 2:**
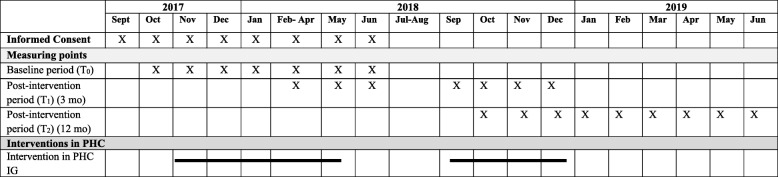
Data collection and intervention programme timeline

*Abbreviations*: *PHC* primary healthcare center, *IG* intervention group, *mo* months

Ethical approval has been provided by the Comitè Ètic d’Investigació (CEI), Fundació Institut Universitari per a la recerca a l’Atenció Primària de Salut Jordi Gol i Gurina (IDIAPJGol), Barcelona, Spain.

### Measures

The selected variables and instruments, as well as the evaluation program timeline, are detailed in Table 3. Data will be imported using an online survey tool and include:

**Table 3:** Outcome measures

Outcome measures	Timeline		
	Pre-intervention	Post-intervention	
		3 mo	12 mo
1. Socio-demographic variables			
Sex, age, marital status, educational level, employment status, reference PHC	X		
2. Diagnostic variables			
Diagnosis depression: MINI	X		
Diagnosis chronic physical illness: ICD-10	X		
Suicide risk: MINI	X		
Cognitive impairment: Pfeiffer questionnaire	X		
3. Clinical variables			
Depressive symptoms: BDI- II questionnaire	X	X	X
Anxiety symptoms: HARS questionnaire	X	X	X
Quality of life: EQ-5D-5 L questionnaire	X	X	X
Pharmacotherapy	X	X	X
Therapeutic compliance: Morinsky-Green test	X	X	X
Feasibility of the intervention	X	X	
4. Blood test and Physical parameters			
Type 2 diabetes mellitus: Analytical HBA1C	X		X
COPD: FEV1 and Medical Research Coincil (mMRC)	X		X
Asthma: Spirometry and Peak flow	X		X
Ischemic heart disease: Analytical cholesterol LDL and Blood pressure	X		X
5. Costs variables			
Pharmacological Treatment	X	X	X
Utilization of services: PHC and hospital	X	X	X
Other comorbidities	X		X
Costs derived from the intervention	X	X	X

*Abbreviations*: *mo* months, *BDI-II* Beck Depression Inventory secondary edition, *COPD* Chronic obstructive pulmonary disease, *EQ* EuroQuol, *FEV1* Forced expiratory volume, *HARS* Hamilton Anxiety Rating Scale, *HBA1C* Hemoglobin A1C test, *ICS-10* International classification of diseases-10th revision, *MINI* Mini international neuropsychiatric interview, *PHC* Primary Healthcare Centers

Diagnostic variables

1. *Diagnosis of depression* according to the MINI interview [27, 28].
2. *Physical diseases* (code CIE-10). Diagnosis of diabetes mellitus (E11-E14 and sub-groups), COPD (J43-J44 and sub-groups), asthma (J45), and/or ischemic cardiopathy (I20-I25 and sub-groups) as registered in the patients’ medical records and confirmed by their family doctor [29].
3. *Risk of suicide* evaluated by the MINI interview [27, 28].
4. *Pfeiffer Scale* to detect cognitive impairment [30, 31]. This is a hetero-administered questionnaire made up of 10 items and scored from 0 to 10 (errors). From 5 onwards an individual is considered to present moderate/severe cognitive decline.

Socio-demographic variables:

Sex, age, civil status, educational level (without studies / incomplete primary ones / completed primary ones / secondary ones / higher education), employment status (employed / housewife / unemployed / disabled / retired). Assigned PHC: rural (doctor’s surgery) / semi-urban (PHC with a population < 15,000 inhabitants) / urban (PHC with a population ≥ 15,000 inhabitants).

Primary outcomes

1. *Clinical remission and/or response of depression* at short and long-term (one year follow-up) of the intervention following Riedel et al. [32]: clinical remission considered as a reduction in the rating < 13 of the BDI-II; response to the intervention as a decrease of at least 50% in the initial evaluation of depressive symptoms according to the BDI-II.

Secondary outcomes

1. *Improvement in the control of the chronic pathology* at 12 months follow-up:

- Diabetes mellitus: controlled with glycosylated hemoglobin, HBA1C (%) [33, 34].
- COPD: lung function measured by post bronchodilator expiratory volume (FEV1). Dyspnea evaluated by the modified score of the Medical Research Council (mMRC), at 5 points [35, 36]
- Asthma: lung function measured by post bronchodilator expiratory volume (FEV1). Determination of peak expiratory flow (PEF) [37].
- Ischemic cardiopathy: measurement of levels of low-density lipoprotein cholesterol and blood pressure [38].

2. *Adherence to medication* is assessed by the *Morinsky-Green* test [39] and the comparison between containers collected in the pharmacy and those prescribed. It will be performed for all the prescribed medication for both groups.
3. *Quality of life* as measured with the *EuroQol (EQ-5D-5 L)* questionnaire. (Original: EuroQol Group 1996. Spanish adaptation: Badia et al. 1999) [40, 41].
4. *Utilization of healthcare services* during the study period. This includes: number of hospital admissions (emergency room and ward) due to complications arising from diabetes, COPD, and ischemic cardiopathy (yes/no), and psychiatric pathology (yes/no); mental health referrals (yes/no) and number; and consultations with the family doctor/nurse (yes/no) and number.
5. *Variables resulting from the intervention with respect to its feasibility.* A questionnaire with 10 items and a Likert scale of 4 categories regarding satisfaction is administered to the patients on completing the intervention. Adherence is assessed through the number of sessions each IG patient attends. An adherence variable is calculated for the intervention: ≥75% attendance or less.

Confounding variables and effect modifiers

1. *Pharmacological treatment.* The defined daily dose for each active ingredient is established during the follow-up period taking into account the number of days, the dosage, and the route of administration. The registered active ingredients belong to the following groups: antidepressants, anxiolytics, antidiabetics (oral, insulin), bronchodilators (selective agonists of Beta2 - adrenergic, anticholinergic), anti-inflammatories: corticosteroids, antihypertensives, and dyslipidemics.
2. *Other associated comorbidities:* cardiac insufficiency, atrial fibrillation, cardiovascular disease, peripheral vascular disease, other chronic diseases.
3. Participation in *other mental health therapies* given by psychologists and psychiatrists.
4. *Anxiety* evaluated with the HARS-Hamilton Anxiety Rating Scale [42].
5. *History of previous depressive episodes* (yes/no).

Cost variables

Expenses included in the cost-effectiveness analysis:

1. *Use of primary care services*: consultation fees according to professional status and level of care.
2. *Use of hospital services*: hospitalization and emergency room consultations.
3. *Cost of psychotropic medication consumed during the study*: anxiolytics and antidepressants according to the current catalogue from the Pharmacist Association.
4. *Derived costs*: training and supervision of the nurses responsible for leading the sessions, their working hours spent with the groups, and expenses related to material used in the sessions and given out to the participants. In order to calculate the cost of the resources 2016 Oblikue rates are employed [43].

### Statistical analysis

The analysis is performed on an intention-to-treat basis. All the patients who have signed the informed consent and attended the initial interview are included. Missing data from the BDI-II evaluation during follow-up will be substituted by the last registered value (Last Observation Carried Forward strategy). At the beginning of the study descriptive statistics will be performed to evaluate the homogeneity between the two groups (IG and CG) with respect to demographic, clinical, and service utilization variables. In order to measure the main objective (rate of remission and response) logistic regression is employed in which the dependent variable is dichotomous (rate of remission and response). The patient’s group is the independent variable. Raw and adjusted odds ratio (OR) are calculated according to age, sex, pharmacologic treatment, and comorbidity. Repeated measure ANOVA analysis will be performed to determine the evolution of the dependent variables during follow-up. For the secondary resulting variables, control of the physical pathologies will be measured by logistic regression in which the dependent variable is dichotomous (control yes/no). The models will be adjusted in the same way as the principal one. Variable differences in the BDI-II rating and other scores with respect to baseline will be calculated. Student’s t test will be employed for the comparison between the groups’ variables and standardized effect size (SES; Kazis et al.1989) [44].

The economic analysis will be carried out with a cost-effectiveness analysis to compare the expenses incurred from the psychoeducation intervention with those from habitual clinical practice over 1 year. The QALYs (quality-adjusted life years) obtained from the EuroQol questionnaire will provide the variable of effectiveness. That of cost will be obtained by measuring all the direct expenses generated by the participants from both groups and including any other related information. For instance, primary care consultations, referrals to specialists, hospitalization, additional tests, analytical tests, and medication expenses. The ratios and incremental costs for the IG versus CG will be calculated. Prices will be based on those of 2016 as the Oblikue database is the most complete for that period. As the healthcare costs will be generated over various years a constant discount rate of 3% per year will be applied. Finally, for the items with the greatest presence in the overall cost, a deterministic sensitivity analysis will be carried out to ensure robustness and reliability of the results obtained [45].

## Discussion

This article describes a study design for investigating the effectiveness of a psychoeducation group intervention carried out by PC nurses. It is aimed at reducing depressive symptoms in individuals with chronic physical disease (diabetes mellitus, COPD, asthma, ischemic cardiopathy) and comparing intervention results with usual clinical practice. A secondary objective is to assess the impact of the intervention on improving control of the physical pathology.

Psychoeducation group interventions have been shown to be effective in reducing depressive symptoms, decreasing the risk of relapse and recurrence, and improving treatment compliance in patients with both depression and an organic, physical disease. In comparison with the general public, depressive patients present a worse control of their physical pathology, less adherence to treatment, and poorer fulfillment of healthy lifestyle habits.

Our results could encourage the implementation of such interventions for patients with depression and chronic organic pathologies within the community, a setting where most of them are attended. If its effectiveness is demonstrated, as a specific, brief intervention that requires relatively few hours of training, it could be carried out by nurses with previous instruction.

A major strength of our work is that the results will be obtained from a naturalistic study as close as possible to the usual clinical practice found in primary care. As a result, it is an intervention that can easily be implemented in such a setting without considerable organizational or structural modifications.

Some limitations include the fact that the nurses who carry out the intervention are aware of the groups the patients belong to. Nevertheless, blinding of some key stages of the study, such as randomization, evaluation of outcomes/confounder variables, and statistical analysis will be maintained. Second, for a long-term follow-up under normal conditions a dropout rate of 25% is expected. In the case of missed appointments and intervention sessions it is hoped that patient adherence will be ensured through telephone calls (at least three times per appointment, one of which is 24 h prior to the appointment) unless the patient has stated reluctance to participate. Third, the patients will present varying pathologies which will increase the heterogeneity of the sample. Nevertheless, most of the patients attended at the PHC present comorbidities and their inclusion represents a greater generalization of results. Fourth, in order to avoid the effect of contamination, patients assigned to the two nurses leading the IG from the same center will be excluded. Fifth, there could be some difficulty in recruiting a sufficient number of patients.

To the best of our knowledge, this is the first study evaluating the effectiveness of a psychoeducation group intervention with patients suffering from both depression and a chronic organic pathology (diabetes mellitus, COPD, asthma, and ischemic cardiopathy) to be carried out by community nurses in a primary care setting.

## Data Availability

All the principal investigators of the study will have access to the complete dataset, and the datasets generated and analysed during the current study will be available from the corresponding author. Results generated during this study will be published in peer-reviewed journals and at national/international congresses. The program has been designed in accordance with CONSORT guidelines, and registration at clinicaltrials.gov will be expected to facilitate transparency and reporting.

## Abbreviations

BDI-II: Beck Depression inventory- Second edition
CG: Control Group
COPD: Chronic obstructive pulmonary disease
IG: Intervention Group
MINI: Mini International Neuropsychiatric Interview
PC: Primary Care
PHC: Primary Healthcare Center

## References

[1] Mathers CD, Loncar D (2006). Projections of global mortality and burden of disease from 2002 to 2030. PLoS Med.

[2] Anderson RJ, Freedland KE, Clouse RE, Lustman PJ (2001). The prevalence of comorbid depression in adults with diabetes: a meta-analysis. Diabetes Care.

[3] Salvador-Carulla L, Bendeck M, Fernández A, Albertí C, Sabes-Figuera R, Molina C, Knapp M (2011). Costs of depression in Catalonia (Spain). J Affect Disord.

[4] Benton T, Staab J, Evans DL (2007). Medical co-morbidity in depressive disorders. Ann Clin Psychiatry.

[5] Rucci P, Gherardi S, Tansella M, Piccinelli M, Berardi D, Bisoffi G (2003). Corsino MA, Pini S. subthreshold psychiatric disorders in primary care: prevalence and associated characteristics. J Affect Disord.

[6] World Health Organization. Depression and other common mental disorders: global health estimates. Geneva: World Health Organization; 2017. Licence: CC BY-NC-SA 3.0 IGO. https://apps.who.int/iris/bitstream/handle/10665/254610/WHO-MSD-MER-2017.2-eng.pdf.

[7] World Health Organization. The global burden of disease: 2004 update. Geneva: World Health Organization; 2008. https://www.who.int/healthinfo/global_burden_disease/2004_report_update/en/. Accessed 27 June 2013.

[8] Nobis S, Lehr D, Ebert DD, Baumeister H, Snoek F, Riper H, Berking M (2015). Efficacy of a web-based intervention with mobile phone support in treating depressive symptoms in adults with type 1 and type 2 diabetes: a randomized controlled trial. Diabetes Care.

[9] National Collaborating Centre for Mental Health. NICE clinical guidelines 91-depression in adults with a chronic physical health problem: recognition and management. London: National Institute for Health and Clinical Excellence; 2009. https://www.nice.org.uk/guidance/cg91. 39808015

[10] Van Dijk S, Pols Alide D, Adriaanse M, Bosmans J, Elders PJM, van Marwijk H, van Tulder M (2013). Cost-effectiveness of a stepped-care intervention to prevent major depression in patients with type 2 diabetes mellitus and/or coronary heart disease and subthreshold depression: design of a cluster-randomized controlled trial. BMC Psychiatry.

[11] Reynolds CF, Cuijpers P, Patel V, Cohen A, Dias A, Chowdhary N, Okereke OI, Dew MA, Anderson SJ, Mazumdar S, Lotrich F, Albert SM (2012). Early intervention to reduce the global health and economic burden of major depression in older adults. Annu Rev Public Health.

[12] Stoop CH, Nefs G, Pommer AM, Pop VJ, Pouwer F (2015). Effectiveness of a stepped care intervention for anxiety and depression in people with diabetes, asthma or COPD in primary care: a randomized controlled trial. J Affect Disord.

[13] Pibernik-Okanovic M, Begic D, Ajdukovic D, Andrijasevic N, Metelko Z (2009). Psychoeducation versus treatment as usual in diabetic patients with subthreshold depression: preliminary results of a randomized controlled trial. Trials.

[14] Pibernik-Okanović M, Hermanns N, Ajduković D, Kos J, Prašek M, Šekerija M, Lovrenčić MV (2015). Does treatment of subsyndromal depression improve depression-related and diabetes-related outcomes? A randomised controlled comparison of psychoeducation, physical exercise and enhanced treatment as usual. Trials.

[15] Schmitt A, Reimer A, Ehrmann D, Kulzer B, Haak T, Hermanns N (2017). Reduction of depressive symptoms predicts improved glycaemic control: secondary results from the DIAMOS study. J Diabet Complicat.

[16] Cuijpers Pim, Andersson Gerhard, Donker Tara, van Straten Annemieke (2011). Psychological treatment of depression: Results of a series of meta-analyses. Nordic Journal of Psychiatry.

[17] Tursi MF, Baes CV, Camacho FR, Tofoli SM, Juruena MF (2013). Effectiveness of psychoeducation for depression: a systematic review. Aust N Z J Psychiatry.

[18] Casañas R, Catalán R, del Val JL, Real J, Valero S, Casas M (2012). Effectiveness of a psycho-educational group program for major depression in primary care:a randomized controlled trial. BMC Psychiatry.

[19] Cuijpers P, Muñoz RF, Clarke GN, Lewinsohn PM (2009). Psychoeducational treatment and prevention of depression: the “coping with depression” course thirty years later. Clin Psychol Rev.

[20] Dalgard OS (2006). A randomized controlled trial of a psychoeducational group program for unipolar depression in adults in Norway (NCT00319540). Clin Pract Epidemiol Ment Health.

[21] Beck AT, Ward CH, Mendelson M (1961). Inventory for measuring depression. Arch Gen Psychiatry.

[22] Beck AT, Steer RA, Garbin MG (1988). Psychometric properties of the Beck depression inventory: twenty-five years of evaluation. Clin Psychol Rev.

[23] Sanz J, Perdigon LA, Vazquez C (2003). Adaptación española del Inventario para la Depresion de Beck G (BDI-II): 2.Propiedades psicométricas en población general. Clin Salud.

[24] Sanz J, Garcia-Vera MP, Espinosa R, Fortun M, Vazquez C (2005). Adaptación española del Inventario para la Depresion de Beck G (BDI-II): 3. Propiedades psicométricas en pacientes con trastornos psicológicos. Clín Salud.

[25] Sanz J, Garcia-Vera MP (2013). Rendimiento diagnóstico y estructura factorial del Inventario de Depresion de Beck-II (BDI-II). Anal Psicol.

[26] Grup de treball del Programa de sessions grupals psicoeducatives per a l’estudi sobre l’efectivitat d’una intervenció grupal psicoeducativa realitzada per infermeres d’atenció primària en pacients amb depressió i comorbiditat física (Estudi PsiCoDep). PI16/01272 i PERIS SLT002/17/00096.

[27] Sheehan DV, Lecrubier Y, Sheehan KH, Amorim P, Janavs J, Weiller E (1998). The Mini-international neuropsychiatric interview (M.I.N.I.): the development and validation of a structured diagnostic psychiatric interview for DSM-IV and ICD-10. J Clin Psychiatry.

[28] Ferrando L, Bobes J, Gibert J. Mini International Neuropsychiatric Interview (M.I.N.I., Version Española 5.0.0 DSM-IV). Madrid: Instituto IAP; 2000. Accesible a través de: http://www.academia.cat/files/425-7297-DOCUMENT/MinientrevistaNeuropsiquatribaInternacional.pdf.

[29] World Health Organization. Guiá de bolsillo de la clasificació n CIE-10 : clasificació n de los trastornos mentales y del comportamiento. Madrid: Editorial Médica Panamericana; 2000.

[30] Pfeiffer E (1975). A short portable mental status questionnaire for the assessment of organic brain deficit in elderly patients. J Am Geriatr Soc.

[31] Martinez de la Iglesia J, Duenas Herrero R, Onis Vilches MC, Aguado Taberne C, Albert Colomer C, Luque Luque R (2001). Adaptación y validación al castellano del cuestionario de Pfeiffer (SPMSQ) para detectar la existenica de deterioro cognitivo en personas mayores de 65 años. Med Clin (Barc).

[32] Riedel M, Möller HJ, Obermeier M, Schennach-Wolff R, Bauer M, Adli M (2010). Response and remission criteria in major depression. A validation of current practice. J Psychiatr Res.

[33] Práctica clínica en la DM2. Análisis crítico de las evidencias por la redGDPS. 2011. http://www.redgdps.org/gestor/upload/file/guias/guia_gedaps_practica-cinica-2010.pdf . Guía de Práctica Clínica sobre Diabetes tipo 2. Guías de Práctica Clínica en el SNS. Ministerio de Sanidad y Consumo 2008.

[34] Casal M, Pinal-Fernández I. Guía de Práctica Clínica de Diabetes tipo 2. Arch Med. 2014;10(2):2. 10.3823/1212, http://www.archivosdemedicina.com/medicina-de-familia/gua-de-prctica-clnica-de-diabetes-mellitus-tipo-2.pdf.

[35] Grupo de trabajo de la Guía de Práctica Clínica para el Tratamiento de Pacientes con Enfermedad Pulmonar Obstructiva Crónica (EPOC). Guía de Práctica Clínica para el Tratamiento de Pacientes con Enfermedad Pulmonar Obstructiva Crónica. (EPOC). Plan de Calidad para el Sistema Nacional de Salud del Ministerio de Sanidad, Servicios Sociales e Igualdad. Unidad de Evaluación de Tecnologías Sanitarias de la Agencia Laín Entralgo; 2012. Guías de Práctica Clínica en el SNS: UETS N° 2011/6. Disponible en: http://www.guiasalud.es/GPC/GPC_512_EPOC_Lain_Entr_resum.pdf.

[36] Guía de Práctica Clínica para el Diagnóstico y Tratamiento de Pacientes con Enfermedad Pulmonar Obstructiva Crónica (EPOC).Guía Española de la EPOC (GesEPOC). Grupo de Trabajo de GesEPOC. Arch Bronconeumol. 2012; 48(Supl 1):2–58. https://www.archbronconeumol.org/es-pdf-S0300289612700352.

[37] GEMA. Guía Española para el Manejo del Asma 2009. http://www.seicap.es/documentos/archivos/GEMA%202009.pdf. 10.1016/S0300-2896(15)32812-X 26707419

[38] Guía de Práctica Clínica de la ESC 2013 sobre diagnóstico y tratamiento de la cardiopatía isquémica estable. Grupo de Trabajo de la Sociedad Europea de Cardiologia sobre diagnóstico y tratamiento de la cardiopatía isquémica estable Rev Esp Cardiol 2014;67(2):135.e1-135e81.

[39] Val Jiménez A, Amorós G, Martínez P, Fernández ML, León M (1992). Estudio descriptivo del cumplimiento del tratamiento farmacológico antihipertensivo y validación del test de Morisky-Green. Aten Primaria.

[40] Group EQ (1990). EuroQol- a new facility for the measurement of health-related quality of life. Health Policy.

[41] Badia X, Roset M, Montserrat S, Herdman M, Segura A (1999). The Spanish version of EuroQol: a description and its applications. European Quality of Life scale. Med Clin.

[42] Lobo A, Chamorro L, Luque A, Dal-Ré R, Badia X, Baró E (2002). Validación de las versiones en español de la Montgomery-Asberg Depression Rating Scale y la Hamilton Anxiety Rating Scale para la evaluación de la depresión y de la ansiedad. Med Clin (Barc).

[43] Oblikue consulting. Disponible en: http://www.oblikue.com/es/plataforma-esalud.html.

[44] Kazis LE, Anderson JJ, Meenan RF (1989). Effect sizes for interpreting changes in health status. Med Care.

[45] Drummond MF, O'Brian B, Stoddart GL, Torrance GW (1997). Methods for the economic evaluation of heathcare programmes.

